# Rhodotorula spp. in the gut – foe or friend?

**DOI:** 10.3205/id000042

**Published:** 2019-09-02

**Authors:** Herbert Hof

**Affiliations:** 1 MVZ Labor Limbach und Kollegen

**Keywords:** mycobiome, fluconazole resistant, gut, Rhodotorula, basidiomyceteous yeast, oleagenous yeast, carotinoids

## Abstract

*Rhodotorula* spp. belong to the basidiomyceteous fungi. They are widespread in the environment. Transmission to humans occur mainly through air and food. Intestinal colonization is rather common, but an overgrowth is normally suppressed, since their optimal growth temperature is exceeded in the body. A massive presence in the gut indicates a disturbance of the balance of the microbial flora due to different causes. One particular reason will be the treatment with azoles because this will create an advantage for these azole resistant fungi. First of all, the finding of increased numbers of *Rhodotorula* in stool specimen is not alarming. In contrast, the colonized human will profit from such a situation since these fungi produce a lot of useful nutrients such as proteins, lipids, folate, and carotinoids. Furthermore, a probiotic effect due to regulation of multiplication of pathogenic bacteria and by neutralizing or destroying their toxins can be anticipated. On the other hand, their massive presence may increase the risk of fungemia and ensuing organ infections especially when the host defense system is hampered. Indeed, *Rhodotorula* spp. range among the emerging fungal pathogens in the compromised host. However, it can be doubted whether all these opportunistic infections reported originate primarily from the gut.

## Introduction

The mycobiome in the human intestinal tract consists of a plethora of various genera and species [1], among them are moulds as well as yeasts. The role of the various moulds remains largely unknown yet whereby the yeast population has gained much more interest until now. Especially the pathogenic role of intestinal *Candida* spp. and *Saccharomyces* spp. has been discussed extensively [2], [3] whereas the relevance of *Rhodotorula* spp. is hardly acknowleged although they are often found in stool specimen of humans [1], [4]. Intestinal colonization in healthy children is found in up to 5% and in young adults even in 12% [5]. Then again, these yeasts have been declared as emerging pathogens inducing fungemia [6], [7], [8], [9] often associated with central catheters [8] and even solid organ infections such as endogenous endophthalmitis [10]. 

## Biology

**Figure 1:**
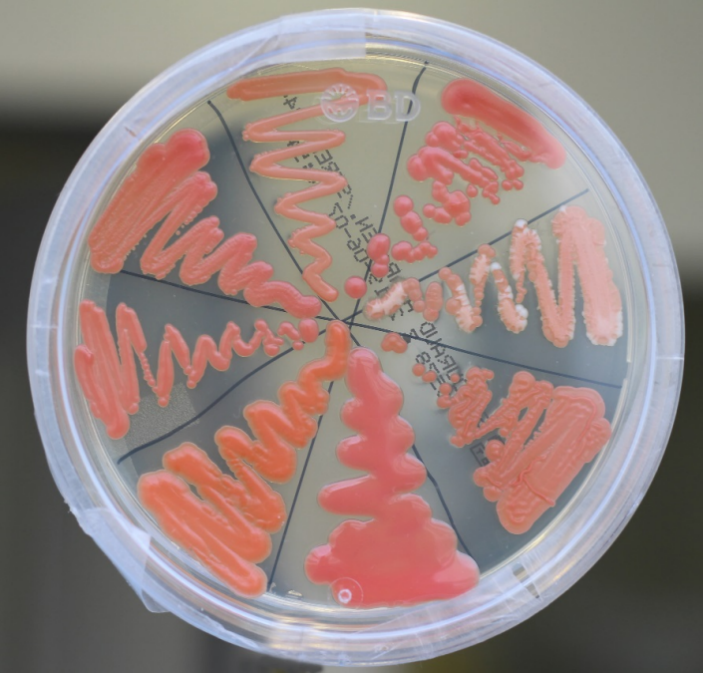
Appearancies of various colonies of Rhodotorula spp. on Sabouraud agar. 5 days after incubation at 30°C. Note the variable colour shades and roughnesses.

In contrast to the ascomycetous yeasts such as *Saccharomyces* spp. and *Candida* spp., *Rhodotorula* spp. belong to the vast phylum of basidiomycetes namely to the family of Sporidiobolales. Typically, *Rhodotorula* spp. produce carotinoids which cause pink to red colonies on agar (Figure 1 (Fig. 1)). (One should be aware that the red colonies of the bacterium Serratia marcescens are due to the pigment prodigiosin). Furthermore, they belong to the oleaginous yeasts characterized by the fact that up to 50% of their dry weight consist of lipids [11]. The optimal temperature for growth is between 18 and 22°C. In vitro, the growth is reduced at temperatures >33°C [12], [13]. The fungus produces unicellular blastoconidia but does not generate pseudohyphae or even hyphae.

Actually, there are 8 species of the genus *Rhodotorula* known and several of them are found in humans for example *R. glutinis*, *R. minuta*, and *R. mucilaginosa*. Since these fungi are tolerant to dryness, they are able to survive in the environment [12]. Hence, this genus can be found almost everywhere in the surroundings, namely in the air – *Rhodotorula* is one of the most common fungi transmitted by air – in soil and water, namely ocean seas as well as lakes, in many kinds of fruits and berries (for example strawberries), milk, toothbrushes and shower curtains. Since these yeasts are endowed with a definite affinity for plastic materials [8], they can be often detected in medical devices, for example on plastic catheters [14], [15] and dental equipments [16] where they will grow in form of biofilms [17]. Furthermore, they colonize often hands of medical staff [8], [18].

Obviously, they are taken up regularly by mammals as well as humans via food, which may lead to intermittent or even permanent gut colonization even in healthy subjects [1], [5], [19] not least because they are relatively resistant to bile [12] in comparison to *Candida albicans* being susceptible to bile acids to some extent [20].

## Antimycotic susceptibilities

Amphotericin B is the most active agent [9]. Furthermore, synergisms between amphotericin B and various non-antifungals have been reported [21]. Flucytosine is also reliably active (Table 1 (Tab. 1)). In general, however, isolates of *Rhodotorula* spp. are resistant to fluconazole and only poorly susceptible to posaconazole and voriconazole [17], [18], [22], [23], [24]. Consequently, these yeasts have a chance to be selected especially in a clinical setting in patients under a longlasting therapeutic regimen with azoles, i.e. fluconazole or posaconazole [25]. Since *Rhodotorula* spp. belong to the basidiomycetes, which quite typically do not contain1-3-β-glucan in their cell wall, they are intrinsically resistant to echinocandins. Hence, colonizations and even breakthrough infections are also reported in patients treated with echinocandins [15].

## Detrimental effects (foe)

### Metabolic disturbances

At least in vitro, *Rhodotorula* spp. like many other oleaginous fungi are enabled to metabolize avidly short-chain fatty acids such as acetic, propionic and butyric acid [26]. Therefore, it could be expected that a massive presence of *Rhodotorula* spp. in the gut could lead to a shortage of those bacterial products. A loss of their beneficial functions, i.e. their anti-inflammatory, anti-oxidative, anti-cancer and antibacterial effects, would be definitely a disadvantage for the host. Furthermore, these short-chain fatty acids are essential for the function of the gut epithelium, liver and brain [27]. However, substantiated data on the influence of *Rhodotorula* spp. on the fatty acid metabolism and composition in the human gut are still lacking.

### Invasion

Especially in the German literature the phenomenon of “persorption”, which means a paracellular translocation of particles and microorganisms including yeasts across the gut wall, has been discussed over centuries [28]. However, the gut epithelium with tight junctions interconnecting the epithelial cells and restricting the passage of particles and microorganisms through the paracellular space represents a solid anatomical barrier inhibiting the unlimited passage of microorganisms. 

There are three main pathophysiological groups of persons where intestinal barrier failure can be definitely observed [29], [30]:

1. in patients undergoing abdominal surgery,
2. in critically ill patients for example cancer patients suffering from mucositis after cytostatic therapy,
3. in patients with chronic pathologic conditions such as HIV infection, liver cirrhosis or inflammatory bowel disease.

Hence, translocation of pathogens from the gut through a dysfunctional mucosal barrier to the mesenteric lymph nodes, the portal vein, and the systemic circulation, eventually leading to sepsis and infectious metastases in various organs, may happen. And even in healthy subjects, this may occur occasionally without any deleterious consequences. A baseline rate of 5–10% of spontaneous translocation of intestinal microorganisms has been estimated to occur in humans [31]. *Rhodotorula* spp. in particular have been suspected to disrupt this barrier to a certain extent [32] presumably facilitating passage. Fungemia [6], [7], [8], [9] and organ infections [10] due to *Rhodotorula* spp. have indeed been described, but it remains obscure, whether in all cases this was directly due to gut colonization [8]. 

Then again, it has been argued [29], [31], [33], [34] that the exposure of the local gut immune system to microbial antigens will have favorable consequences since it may strengthen the defense system of the host in case of a massive invasion and may help to establish tolerance to antigens of commensal microflora. 

### Allergy

It has been discussed that several yeast antigens, for example mannans, mannoproteins as well as several enzymes such as enolase, in the gut may stimulate the intestinal and furthermore the systemic immune system leading to hypersensitivity against inhaled as well as ingested antigens [35], [36], [37], [38]. Since *Rhodotorula* spp. also generate those constituents, it could be speculated that they should be able to induce the same hypersensitivities, too.

## Beneficial effects (friend)

### Fungal products as nutrients

*Rhodotorula* spp. like many other yeasts may accumulate or secrete proteins and various enzymes [11] such as amylase, cellulase, xylanase etc. contributing to the degradation of the viscosity-generating soluble fibers in food items, considered to be nutritionally advantageous [39]. Fungal proteases have been shown to degrade also bacterial toxins [40]. Furthermore, *Rhodotorula* is a good producer of folate [39] which is an important micronutrient for all living organisms.

In addition, these oleaginous yeasts are endowed with a particular capacity to form saturated long-chain fatty acids like oleic, linoleic, and in particular palmitic acids, which can be stored in the fungal cells, so that up to 50% of the dry weight of these cells consist of lipids [11]. It remains still obscure actually what this fact finally means for the host. Whereas the role of the bacterial part of the microbiome on the lipid metabolism has been elucidated recently [41], the influence of fungi and especially of oleaginous yeasts such as *Rhodotorula* has been ignored until now. Hence, it can only be speculated whether the saturated fatty acids represent a high calorie source ensuing a nutritional benefit or whether it may support in some cases obesity. 

One characteristic trait of *Rhodotorula* spp. is the production of different carotinoids giving the fungal colonies their pink appearance. These fungal metabolites can be further converted into vitamin A, which by the way cannot be produced by a human host himself [1]. This vitamin is well known for its protective effects on epithelia [42]. Consequently, it could be expected that it may help to strengthen the intestinal barrier function protecting against bacterial translocations and infections.

By the way, in the future, recombinant strains of *Rhodotorula* spp. may eventually be used as living cellular carriers delivering various biologically active agents such as insulin into the gut as has been shown in animal experiments [43]. 

### Probiotic effect

*Saccharomyces* spp. and in particular the so-called *S. cerevisiae* *var. boulardii* are used therapeutically in patients with diarrhoea caused by *Clostridium difficile*, enterohemorrhagic *E. coli* or *Salmonella* [44], [45]. At least partially, the positive effects are due to competition with the intestinal flora [40] and especially by binding of pathogenic bacteria or their toxins to the mannan structures in the cell surface of yeasts [46], [47]. In principle, *Rhodotorula* spp. express similar molecules in their cell wall, and thus, it could be expected that the commensal *Rhodotorula* spp. also can exert similar beneficial effects. Furthermore, *S. boulardii* has the potential to interact with the intestinal immune system and to suppress the intestinal inflammation by inhibiting the migration of inflammatory leukocytes from the mesenteric lymph nodes [44]. Then again, not all properties of *S. cerevisiae* can be replaced by *Rhodotorula* spp. Whereas oral feeding of mice with living *S. cerevisiae* stimulated the intestinal epithelium to produce purines which leads to elevated uric acid blood levels above controls, *Rhodotorula* did not [32]. 

## Evaluation

Although the mycobiome of the gut is numerically definitely smaller than the bacterial community, it may exert a disproportional effect on health or disease [1]. Prolonged treatment with broad-spectrum antibiotics especially with those secreted into the gut such as ciprofloxacin and ceftriaxone can disturb the balance in favor of fungi [43] especially of yeasts and among them *Rhodotorula* spp. belonging to the basidiomycetes. The additional therapy with metronidazole, eliminating the anaerobic bacteria will further enhance the fungal overgrowth [48]. An antagonism between bacteria such *E. coli* and *Rhodotorula* in particular has been observed already long time ago [12]. Whereas in older publications [12] it has been reported, that *Rhodotorula* are cultured in stool specimen rather rarely, today yeasts of this genus are the most common yeasts in stool specimen besides *Saccharomyces* spp. and *Candida* spp. [5], [19], [32]; indeed the numbers detected are quite considerable in some cases. It can be argued that the frequent use of fluconazole and echinocandins, respectively, for therapy and prophylaxis of yeast infections may have contributed to this fact, since *Rhodotorula* spp., which are intrinsically resistant to these antimycotic agents (Table 1 (Tab. 1)), will profit from such a selective pressure. The overgrowth of *Rhodotorula* spp. in the gut occurs in spite of non-favorable conditions. Because the optimal growth temperature for *Rhodotorula* spp. is between 28 and 22°C [12], [13] and the multiplication rate is limited at temperatures >33°C, in normal, healthy individuals the number of *Rhodotorula* spp. in the gut will be restricted. The fact that increased numbers of *Rhodotorula* spp. in stool specimen of sick patients after various medical interventions are found rather often [1], [4], [8] indicates, however, a definite alteration of the milieu and a disturbed balance in the microbial population in the gut of those individuals. Obviously, these fungi can survive and multiply in the extreme, unfavorable conditions in the gut. A relative resistance to bile acids [12] may facilitate the colonization.

The question here is, which role plays the fungal flora in the gut. It is well accepted that commensal, low-virulent fungi in the gut like commensal bacteria are able to induce disease at least in case that the individuals has developed a considerable degree of predisposition [44]. 

It has been argued that Candida spp. in the gut are relatively often associated with disease, whereas *Rhodotorula* spp. are not [49]. Although one can assume that *Rhodotorula* spp. like other microorganisms in the gut may have the chance to cross a dysfunctional intestinal barrier, it remains still uncertain whether this pathway represents really the primary cause of fungemia [8]. Indeed, it can be doubted whether the pathogens found in blood or in solid organs originate from the population found in the stool specimen, since it has to be kept in mind that the ubiquitous and saprophytic *Rhodotorula* spp. are transmitted rather frequently by water [8], [50] and especially by air [8]. Hence, at least transient colonization not only of the gut but also of other sites such as the skin occurs, which might become subsequently the main source of catheter infections and ensuing fungemia. Most cases of *Rhodotorula* fungemia are actually associated with contaminated central catheters [6], [7], [8], [9] especially in patients with cytotoxic drugs [3]. Potential pathogenic property of *Rhodotorula* spp. has long been known and experimental infections of animals are reported [8], [51]. In the last 3 decades it is becoming more and more evident, that *R. glutinis*, *R. minuta* and *R. mucilaginosa* range among the emerging pathogens especially in immunocompromised patients provided with central venous catheters, since *Rhodotorula* spp. are able to settle on surfaces of implanted materials and to form biofilms there [8]. In comparison to the high prevalence of Candida spp. in blood cultures the frequency of *Rhodotorula* spp., however, remains still relatively low [8].

Yeasts in the gut are not only innocent commensal bystanders [44]. They may induce not only infections, but they may trigger metabolic disturbances [4], [19] and engender allergy [35], [36], [37], [38]. It can be assumed that *Rhodotorula* spp. exert at least partially similar effects [26].

Then again, a host will profit to some extent from *Rhodotorula* spp. in his gut, since these yeast produce nutrients such as lipids, carotinoids, folate and proteins [1], [11], [39]. In addition, fungal enzymes may help to digest vegetable food [39]. A probiotic effect may be exerted by binding [46], [47] or destroying bacterial toxins for example those from *C. difficile* [40]. However, the probiotic effect of *Rhodotorula* may be lower than that of Saccharomyces and other ascomycetic yeasts, since *Rhodotorula* spp. like all basidiomycetes do not possess 1-3-β-glucan in their cell wall, so that these fungi will not be able to trigger the dectin 1-receptor on the surface of macrophages. Consequently, it can be anticipated that a stimulation of the local immune system will not happen.

Now, overall *Rhodotorula* spp. obviously will have certain beneficial impacts on the health of colonized humans; hence an eradication is not indicated in every case. But on the other hand these opportunistic fungi are principally able to induce serious infections at least in the compromised patient. Thus, a definitive assessment whether *Rhodotorula* spp. are foes or friends is actually not yet possible, since the knowledge about this neglected and underestimated yeast is still incomplete. Hence, some of the above remarks will possibly not be maintained over time.

## Conclusion

*Rhodotorula* spp. belong to the basidiomycetic fungi. They are widespread in the environment. Transmission to humans occur mainly through air and food. Intestinal colonization is rather common, but an overgrowth is normally suppressed, since their optimal growth temperature is exceeded in the body. A massive presence in the gut indicates a disturbance of the balance of the microbial flora due to different causes. One particular reason will be the treatment with azoles, because this will create an advantage for these azole resistant fungi. First of all, the finding of increased numbers of *Rhodotorula* in stool specimen is not alarming. In contrast, the colonized human will profit from such a situation, since these fungi produce a lot of useful nutrients such as proteins, lipids, folate and carotinoids. Furthermore, a probiotic effect due to regulation of multiplication of pathogenic bacteria ad by neutralizing or destroying their toxins can be anticipated. On the other hand, their massive presence may increase the risk of fungemia and ensuing organ infections especially when the host defense system is hampered. Indeed, *Rhodotorula* spp. range among the emerging fungal pathogens in the compromised host. It can be doubted, however, whether all these opportunistic infections reported originate primarily from the gut.

## Notes

### Competing interests

The author declares that he has no competing interests.

## Figures and Tables

**Table 1:**
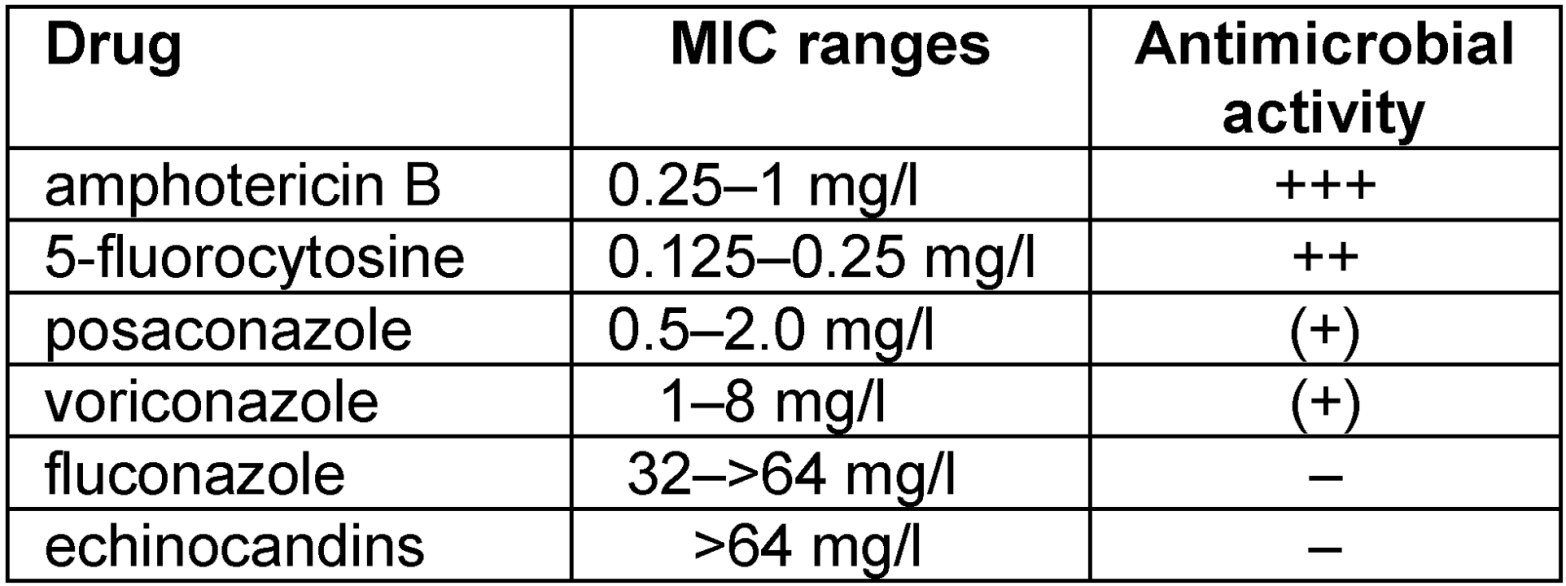
Antimicrobial susceptibilities of Rhodotorula spp. (according to [22])
